# Bacteriuria in Paediatric Oncology Patients: Clinical Features, Distribution and Antimicrobial Susceptibility of Bacterial Pathogens at University Hospital Centre Zagreb, Croatia over a 4-Year Period

**DOI:** 10.3390/antibiotics13020118

**Published:** 2024-01-25

**Authors:** Nina Predavec, Antonio Perčinić, Zoran Herljević, Violeta Rezo Vranješ, Maja Pavlović, Zrinko Šalek, Tomislav Kuliš, Ernest Bilić, Ivana Mareković

**Affiliations:** 1 Department of Clinical Microbiology, Infection Prevention and Control, University Hospital Centre Zagreb, 10000 Zagreb, Croatia; 2 Division of Pediatric Hematology and Oncology, Department of Pediatrics, University Hospital Centre Zagreb, 10000 Zagreb, Croatia; 3 Department of Urology, University Hospital Centre Zagreb, 10000 Zagreb, Croatia; 4 School of Medicine, University of Zagreb, 10000 Zagreb, Croatia

**Keywords:** urinary tract infection, bacteriuria, pediatric oncology, antibiotics

## Abstract

Bacteriuria in paediatric oncology patients have not been well studied. This retrospective study analysed clinical features, distribution and antimicrobial susceptibility of bacterial pathogens cultured from urine in paediatric oncology patients over a 4-year period (2019–2022). A total of 143 episodes of bacteriuria were documented in 74 patients. Neutropenia was present in 17.5% (25/143), symptoms in 25.9% (37/143) and urinary catheter in 7.0% (10/143) episodes. Symptomatic bacteriuria episodes were statistically significantly more frequent in patients with neutropenia (*p* = 0.0232). The most common bacterial pathogens were *Escherichia coli* (n = 49; 32.2%), *Klebsiella* spp. (n = 34; 22.4%), *Pseudomonas aeruginosa* (n = 22; 14.5%) and *Enterococcus* spp. (n = 21; 13.8%). Extended-spectrum β-lactamases-producing (ESBL) Enterobacterales were found in 11 episodes (11/143; 7.7%) with the highest proportion among *Klebsiella pneumoniae* isolates (n = 7/34; 20.6%). No carbapenem-resistant Enterobacterales, multidrug-resistant *P. aeruginosa* or vancomycin-resistant *Enterococcus* spp. were found. The most important novelties are demonstrating *P. aeruginosa* as one of the prominent bacteriuria pathogens in this patient population, presence of ESBL isolates and carbapenem-resistant *P. aeruginosa* later during hospitalization highlights the need for appropriate antimicrobial treatment. However, because of the small number of symptomatic patients, further studies are needed to clarify the importance of including urine culture in the diagnostic process in patients with febrile neutropenia.

## 1. Introduction

Urinary tract infection (UTI) is characterized by the invasion and replication of pathogens in the urinary tract. The majority of UTIs occur in the lower urinary tract while a minority results in pyelonephritis [1]. Two percent of boys and eight percent of girls will be affected by UTI in the first seven years of life [2,3]. Additionally, UTI is the second most common bacterial infection in children [3,4,5]. Risk factors that predispose children to UTI include female sex, age of two to six years, bladder and bowel dysfunction and vesicoureteral reflux [6,7]. Girls are more susceptible to UTI because of the shorter length of the female urethra and the regular heavy colonization of the perineum by enteric organisms [8].

Bacteriuria in paediatric oncology patients with solid tumours or haematological diseases has not been well studied. Treatment of these patients often includes chemotherapy which can lead to neutropenia and a higher risk of bacterial infection [9]. According to some authors, UTIs are as common as bacteraemia in paediatric oncology patients with febrile neutropenia. In contrast, a recent study found that UTI was an uncommon cause of infection in these patients. However, it should be noted that studies investigating bacteriuria in this patient group are rare. Unlike acute respiratory or gastrointestinal infections, UTIs may present with nonspecific symptoms and no obvious clinical signs other than fever. Therefore, it may be missed unless urine cultures are routinely performed [10,11].

Asymptomatic bacteriuria is defined as the presence of one or more bacterial species growing in the urine at a specific quantitative level, regardless of the presence of pyuria, in the absence of signs or symptoms due to UTI [12]. Although asymptomatic bacteriuria is common and the treatment is generally not recommended, little information is available on the incidence of asymptomatic bacteriuria in paediatric oncology patients [13].

The most common bacterial pathogens that cause UTIs are enteric bacteria, including *Escherichia coli*, *Klebsiella* spp. and *Proteus* spp. [14]. UTI caused by *Proteus mirabilis* occurs more often in boys than in girls [15,16]. Multidrug-resistant bacteria (MDRB) have become an increasing problem in the treatment of UTIs in children [17]. In recent years, the emergence of extended-spectrum β-lactamases (ESBL)-producing organisms has led to increasing antimicrobial resistance [18]. The emergence of these organisms is facilitated by the use of broad-spectrum antibiotics, especially third-generation cephalosporins and quinolones [19]. Other risk factors of ESBL-associated UTIs in children include UTI prophylaxis, previous UTIs, hospitalization within the past three months, recent antibiotic use and high UTI recurrence rate [20,21,22]. The growing problem also includes carbapenem-resistant Enterobacterales as well as carbapenem-resistant *Pseudomonas aeruginosa* and vancomycin-resistant enterococci due to limited treatment options.

Due to the lack of information on bacteriuria in paediatric oncology patients, we retrospectively analysed the clinical characteristics, the distribution and the antimicrobial susceptibility of bacterial pathogens cultured from urine in paediatric oncology patients over a 4-year period (2019–2022).

## 2. Materials and Methods

This study is a single-centre retrospective case-control study of paediatric patients with solid tumours and haematological diseases admitted to the University Hospital Centre Zagreb, a 1510-bed tertiary care hospital, between January 2019 and December 2022 and experiencing bacteriuria. In addition to the distribution of bacterial pathogens and their antimicrobial susceptibility, asymptomatic and symptomatic bacteriuria episodes were compared according to the patient’s characteristics and bacterial pathogens. According to the University Hospital Centre Zagreb’s Ethics Committee’s research approval (2 October 2023 Klasa: 8.1-23/207-2 Broj: 02/013AG), informed consent was not required. Patients with bacteriuria episodes with more than 10^4^ CFU/mL of bacteria in urine culture were included. Urine samples were collected by mid-stream urine or from a previously disinfected urinary catheter port. One episode of bacteriuria was considered distinct from another when the time interval between urine culture samples was more than two weeks. Urine samples were collected from (a) patients with suspected UTI and (b) surveillance cultures (together with nasopharyngeal and throat swabs, and stool samples) routinely obtained in paediatric oncology patients at our centre at admission and once a week. 

Identification of bacteria grown from the urine culture was performed with matrix-assisted laser desorption/ionization time-of-flight mass spectrometry (MALDI-TOF MS) (Bruker Daltonics GmbH, Bremen, Germany) to the species level. Antimicrobial susceptibility was tested by disc diffusion method according to the European Committee on Antimicrobial Susceptibility Testing (EUCAST) guidelines [23]. For antimicrobial agent combinations for which EUCAST does not provide breakpoints, Clinical and Laboratory Standards Institute (CLSI) breakpoints were used. These combinations included the interpretation of piperacillin/tazobactam and ampicillin/sulbactam for *Acinetobacter* spp. [24]. In Gram-negative carbapenem-resistant isolates, susceptibility to imipenem and meropenem as well as to colistin was tested additionally with broth microdilution method performed according to EUCAST standards [25]. The presence of carbapenemases in Enterobacterales isolates was initially detected by immunochromatographic assay (RESIST-5 O.O.K.N.V., Gembloux, Belgium). The genes conferring resistance to β-lactams including class A KPC, class B metallo-β-lactamases (blaVIM and blaNDM) and carbapenem hydrolysing oxacillinases (blaOXA-48-like) were determined by multiplex PCR as previously described [26]. For the antimicrobial susceptibility analysis, intermediate susceptible isolates were considered susceptible. Multidrug resistance was defined as resistance to three or more classes of antimicrobial agents [27].

Data was collected from the laboratory information system (LIS) of the Department of Clinical Microbiology, Infection Prevention and Control with regards to the total number of positive urine cultures per patient, type of bacterial pathogen and antimicrobial susceptibility. The following information was accessed from medical records in the hospital information system: patients’ demographics, presence of a urinary catheter, history of UTI, white blood cell (WBC) count and presence of one of the following symptoms of UTI: fever, dysuria, back and/or suprapubic pain and other symptoms (lack of appetite, irritability, nausea, vomiting and diarrhea). WBC counts below 1.0 × 10^9^/L, on the day when the first positive urine culture during the episode was obtained, suggested neutropenia.

All reported data except the age of patients is categorical and it is reported as counts and percentages. To check for statistically significant differences between groups of symptomatic and asymptomatic bacteriuria episodes, depending on the number of occurrences, we have used Chi-square tests or Fisher’s exact test. Additionally, to examine the association between different causative pathogens and symptomatic bacteriuria, we have used the Fisher-Freeman-Halton exact test. A significant two-sided *p*-value was defined as *p* < 0.05. For the statistical analyses, we have used MedCalc Statistical Software version 20.0.4 (MedCalc Software Ltd., Ostend, Belgium) and for the Fisher-Freeman-Halton test, we have used IBM SPSS Statistics for Windows, Version 27.0.1.0 (IBM Corp, Armonk, NY, USA).

## 3. Results

During 4 years, 143 bacteriuria episodes were recorded in 74 paediatric patients with malignancies. Thirty-seven (37/74; 50.0%) patients had only one episode, whereas multiple episodes occurred in the same number of patients with a maximum of six episodes per patient. Out of the total of 143 episodes, 134 (134/143; 93.7%) were monomicrobial and nine (9/143; 6.3%) polymicrobial with a maximum of two bacterial pathogens detected in the same urine culture. The clinical and demographic characteristics of the study patients are shown in Table 1. Males accounted for 47.3% (35/74) of the patients and the median age was 8 years (range 0–17 years). Haematological malignancies represented the most common underlying malignancy (63/74; 85.1%) compared with 11 (11/74; 14.9%) paediatric patients with solid tumours. Acute lymphoblastic leukaemia (ALL) was the most common haematological malignancy (45/74; 60.8%) and neuroblastoma (4/74; 5.4%) was the most common solid tumour. A total of 20 (20/74; 27.0%) bacteriuria episodes occurred in neutropenic children.

Thirty-seven bacteriuria episodes (25.9%) were symptomatic. Comparison of symptomatic and asymptomatic bacteriuria episodes is shown in Table 2. Symptomatic bacteriuria episodes were more frequent but not statistically significant in patients with solid tumours, (*p* = 0.3997), a history of UTI (*p* = 0.4804), a urinary catheter (*p* = 0.2919) as well as in patients infected with *Klebsiella* spp., *Proteus mirabilis* and polymicrobial bacteriuria (*p* = 0.1641). As expected, symptomatic bacteriuria episodes were statistically significantly more frequent in neutropenic patients (*p* = 0.0232).

A total of 152 bacterial isolates were recovered from 143 bacteriuria episodes. The majority of isolates were Gram-negative bacteria (86.2%; 131/152). The most frequently isolated bacterial pathogen was *Escherichia coli* (49/152; 32.2%), followed by *Klebsiella* spp. (34/152; 22.4%) and *Pseudomonas aeruginosa* (22/152; 14.5%). *Enterococcus* spp. (21/152; 13.8%) was the only and the most common Gram-positive pathogen. Nine polymicrobial bacteriuria episodes with two pathogens simultaneously detected were documented; they were caused by *Klebsiella* spp. and *P. aeruginosa* (3/9; 33.3%), *Klebsiella* spp. and *E. coli* (2/9; 22.2%), *E. coli* and *Enterococcus* spp. (2/9; 22.2%), *E. coli* and *P. aeruginosa* (1/9; 11.1%) and two different members of *Klebsiella* spp. (*Klebsiella pneumoniae* and *Klebsiella oxytoca)* (1/9; 11.1%) (Table 3). 

We analyzed the time from the date of admission to the positive urine culture in which a specific pathogen was isolated (Table 4). *E. coli*, *Enterococcus* spp., *Proteus mirabilis* and *Citrobacter* spp. were isolated within shorter periods (median 8, 7, 10 and 1 day, respectively) while *Klebsiella* spp., *P. aeruginosa* and *Acinetobacter* spp., *E cloacae* and *A. junii* (median 28.5, 17.5, 21 and 69 days, respectively) within longer periods after admission. 

We analysed in vitro antimicrobial susceptibility in 152 bacterial isolates recovered from positive urine cultures. Table 5 shows a detailed description of antimicrobial resistance among Gram-negative isolates. Among narrow-spectrum antimicrobials the highest resistance rate was to ampicillin (88.0%; 95/108) and the lowest to co-amoxiclav (16.7%; 18/108). Moreover, *E. coli* and *Proteus mirabilis* showed no resistance to co-amoxiclav. In general, imipenem, meropenem and amikacin were the most effective in vitro antimicrobial agents for Gram-negative bacteria with the lowest resistance rates (1.5%, 1.5%, and 0.8%). Overall resistance rates to piperacillin/tazobactam and cefepime were also low (<10%), although higher resistance rates to these two antimicrobial agents were detected in *Enterobacter cloacae* (33.3% and 22.2%, respectively) and *Klebsiella* spp. (14.7% for both antimicrobial agents). There were no resistant *P. aeruginosa* isolates to cefepime and 4.6% were resistant to piperacillin/tazobactam. 

ESBL-producing Enterobacterales were found in 11 episodes (11/143; 7.7%) with the highest proportion among *K. pneumoniae* isolates (7/34; 20.6%) followed by ESBL-producing *E. cloacae* (1/9; 11.1%) and *E. coli* (3/49; 6.1%). No carbapenem-resistant Enterobacterales (CRE) or multidrug-resistant *P. aeruginosa* isolates were detected. Carbapenem resistance was detected only in two *P. aeruginosa* isolates that were susceptible to colistin. Susceptibility to ceftolozane/tazobactam was tested in fifteen *P. aeruginosa*, one *E. cloacae* and one *K. pneumoniae* isolate with only one *K. pneumoniae* isolate being resistant. Resistance to ceftazidime/avibactam in three isolates tested was not detected. One *P. aeruginosa* isolate that was tested for imipenem/relebactam was resistant. All 11 ESBL-producing isolates were detected in patients with haematological malignant diseases—eight in patients with ALL, two in patients with Hodgkin lymphoma and one in patients with AML. Two carbapenem-resistant *P. aeruginosa* isolates were also detected in patients with haematological malignant diseases—non-Hodgkin lymphoma and ALL. No multidrug-resistant isolates were detected in patients with solid tumours.

Among Gram-positive pathogens, 17 bacteriuria episodes (17/143; 11.9%) were caused by *E. faecalis* and 4 (4/143; 2.8%) by *E. faecium*. All *E. faecalis* isolates were susceptible to ampicillin. No vancomycin-resistant *Enterococcus* spp. (VRE) or methicillin-resistant *Staphylococus aureus* (MRSA) was found. 

## 4. Discussion

Bacteriuria has been not well studied in pediatric oncology patients. In adult oncology patients with febrile neutropenia (FN), UTI is diagnosed in 5% to 30% of this patient population. However, views vary widely—from studies supporting the inclusion of urine culture in the diagnostic workup of febrile neutropenia patients to studies questioning this investigation due to its lack of utility and cost-effectiveness. The controversy is even more evident in pediatric oncology patients lacking comprehensive data [10,11,28]. Therefore, we conducted a study involving 143 bacteriuria episodes in 74 pediatric oncology patients and their clinical and microbiological characteristics to clarify this issue. 

In this study, the most common pathogen in bacteriuria was *E. coli*, followed by *Klebsiella* spp., *P. aeruginosa* and *Enterococcus* spp. In several studies analyzing pediatric oncology patients, the distribution of pathogens was different. While in all studies *E. coli* was the predominant pathogen, the distribution of other bacterial genera differs. For example, *P. aeruginosa* was completely absent in some studies [28]. In contrast, a retrospective study of the incidence and causative organisms of bacteriuria in children with cancer suggested that *P. aeruginosa* is more commonly found as a causative pathogen of bacteriuria in children with cancer compared to healthy children [29]. Similar to other studies including children with cancer, in our study, *P. aeruginosa* was the third most common bacterial pathogen [30]. These results imply that *P. aeruginosa* may be an important pathogen responsible for UTIs in children with cancer. *P. aeruginosa* is often considered a cause of complicated and healthcare-associated UTIs [31]. Previous UTI (74/143; 51.7%) requiring antimicrobial treatment and the presence of a urinary catheter (10/143; 7.0%) may have contributed to the increased proportion of *P. aeruginosa* in bacteriuria episodes in our study. 

We described the isolation of *E. coli*, *Enterococcus* spp., *Proteus mirabilis* and *Citrobacter* spp. from urine culture earlier during hospitalization. Additionally, *Klebsiella* spp., *P. aeruginosa* and *Acinetobacter* spp., *E cloacae* and *A. junii* were isolated later during hospitalization. This finding can be helpful in the choice of antimicrobial agent in the treatment of UTI—after the second week, *K. pneumoniae* ESBL and *P. aeruginosa* should be covered by empirically administered antimicrobial agents for UTI. 

We describe low antimicrobial resistance to antimicrobial agents in both, Gram-positive and Gram-negative isolates. In so far conducted studies about bacteriuria in pediatric cancer patients, antimicrobial resistance rates varied. In our study ESBL-producing Enterobacterales were found in 11 episodes (11/143; 7.7%) with the highest proportion among *K. pneumoniae* (7/34; 20.6%) but in <10% of *E. coli* isolates (3/49; 6.1%). Therefore, ESBL isolates should be included in antimicrobial treatment decision-making, especially when *K. pneumoniae* isolates are detected. In other studies, the proportion of ESBL-producing isolates varied from low (7% in *E. coli* and 7% in *K. pneumoniae*) to high (37% in Gram-negative organisms) [29,30]. There were no resistant *P. aeruginosa* isolates to cefepime and 4.6% were resistant to piperacillin/tazobactam in our study. Carbapenem resistance was only detected in 9.1% of *P. aeruginosa* isolates (2/22) and no carbapenem-resistant Enterobacterales were detected. No VRE and MRSA were found among Gram-positive isolates.

Interestingly, resistance rates in our hospital and Croatian nationwide surveillance were greater than what we found in our patients’ bacteriuria isolates. There was a change in the percentage of VRE among *E. faecium* isolates in the period 2019 to 2022 from 32.0% to 42.0% at the Croatian national level and from 41.4% to 36.9% at the hospital level. At the Croatian and hospital levels, the proportions of carbapenem-resistant *K. pneumoniae* changed from 6.0% to 13.0% and from 7.3% to 18.7% to, respectively [32,33]. We have observed an excellent level of susceptibility to meropenem among Gram-negative isolates with only 1.5% of isolates showing resistance. The resistance rates to cefepime and piperacillin/tazobactam were both below 10%. Despite the increasing prevalence of resistant strains in both, the general patient population and cancer patients, these strains remain uncommon in certain hospital wards or localities. Therefore, it is crucial to have careful surveillance and knowledge of the local epidemiology at the regional, national, hospital, and hospital ward levels. Interestingly, most bacterial surveillance data do not differentiate between the rates of antibiotic resistance in adult patients and children. Research has shown that antimicrobial resistance may vary depending on the age of the patient [34]. Differences in antimicrobial resistance prevalence may be due to differential antimicrobial stewardship practices and infection prevention measures. Furthermore, our data and observations suggest that empirical antibiotic therapy for pediatric hematology/oncologic patients cannot be directly extrapolated from the resistance rates observed in adult patients.

Interestingly, ESBL isolates and carbapenem-resistant *P. aeruginosa* were found in pediatric patients with haematological diseases and not in patients with solid tumours. This can be associated with different types of cancer treatment administered in these patients, level of immunosuppression, and administered prophylaxis. 

In many haematological centres, colonization status is evaluated in high-risk neutropenic patients by the performance of surveillance cultures every week. The increased permeability of the intestines, caused by cytotoxic antineoplastic therapy, often leads to the translocation of colonizing bacteria, resulting in invasive infections in patients with haematological malignancies [35]. In a study by de la Court et al., the majority of bacteraemia caused by third-generation cephalosporins-resistant Gram-negative bacteria was preceded by colonization [36]. In a study by Torres et al. surveillance cultures of nasal, pharyngeal, axillary and rectal swabs were performed at admission and subsequently every week [37]. The researchers found that surveillance cultures, particularly from axillary-rectal sites, were valuable in predicting the occurrence of bloodstream infections caused by multidrug-resistant bacteria (MDRB) and guiding empirical antibiotic treatment. A study by MacFadenn et al. found that a patient’s prior urine culture results are useful in predicting the identity and susceptibility of a current positive urine culture [38]. 

In our research, we found that 25.9% of bacteriuria cases were symptomatic. These symptomatic cases were more common in patients with solid tumours, a history of urinary tract infections, and urinary catheters, as well as in patients with *Klebsiella* spp., *P. mirabilis*, and polymicrobial bacteriuria. However, we observed a statistically significant increase in symptomatic bacteriuria only in patients with neutropenia. Similarly, in studies investigating bacteriuria in paediatric oncology patients conducted by Mitsuboshi et al. and Hirmas et al., 41.0% and 43.0% of bacteriuria episodes were symptomatic, respectively, although, there was no significant difference in characteristics between children with asymptomatic and those with symptomatic bacteriuria [29,30]. Accurate diagnosis of UTI is important since it can prevent the spread of infection and recurrent UTI can contribute to scarring, which may lead to hypertension and renal failure. It is worth mentioning that in neutropenic paediatric oncology patients with UTI, pyuria can be absent and the leucocyte esterase urine test has limited sensitivity. Also, studies have shown that the nitrite urine test is less effective in younger than in older patients [30]. 

We do not suggest all asymptomatic paediatric oncology inpatients undergo routine urine culture. According to our findings, symptomatic bacteriuria was statistically significantly more frequent in neutropenic patients. This finding is an important addition to the current knowledge because the opinions about the role of UTI in febrile neutropenia so far been conflicting—from authors who think that the urinary tract may be a common site of infection in paediatric oncology patients with neutropenia to the authors who think that UTI is an infrequent cause of infection in these patients and that urinalysis is indicated only in children with febrile neutropenia with urinary signs/symptoms and in asymptomatic patients with a history of urinary tract disease or unknown history [10,11,28]. Even yet, our results did not resolve the current problem, given the small number of patients (n = 37) in our study who had symptomatic bacteriuria. More study on bacteriuria in paediatric oncology/haematology patients is necessary due to the paucity of papers on the subject. Until then, current guidelines are accessible; The International Paediatric Fever and Neutropenia Guideline Panel states that urine culture and urinalysis can be evaluated in patients who have an easily available clean-catch, mid-stream specimen [34]. 

Our study has several limitations. First, our investigation was limited to one hospital centre, which makes it challenging to generalize the results to all children with cancer diseases. That might, however, serve as motivation for other facilities to carry out comparable research to examine their bacterial pathogens and resistance rates in paediatric oncology/haematology patients. Second, only hospitalized patients were included in the study; the frequency and characteristics of bacteriuria in hospitalized patients may differ from those seen in outpatients. Because the hospital environment typically houses a higher concentration of multidrug-resistant organisms, separate insight into hospital patients is advantageous. Third, the study did not provide comprehensive data on the susceptibility of the isolates to newer antimicrobial agents (i.e., new beta-lactam/beta-lactamase inhibitor combinations) cefolozane/tazobactam, cefazidime/avibactam and imipenem/relebactam). The number of isolates tested for these new antimicrobials is low as we routinely test for their susceptibility only when there is resistance to the first and second-line antimicrobial agents. For our patients, information about resistance rates of novel antimicrobial agents is still not critical due to the low level of resistance to the “old” ones.

Despite the limitations, our findings provide valuable insights into bacteriuria in children with solid tumours and haematological disorders. It is common for paediatric oncology patients to have asymptomatic bacteriuria, with *P. aeruginosa* being one of the top three bacterial pathogens in these cases. At our centre, it is important to consider ESBL isolates in the decision-making process for antimicrobial treatment, especially when *K. pneumonia* isolates are detected. For UTIs, empirically administered antimicrobial agents should cover *K. pneumoniae* ESBL and *P. aeruginosa* after the second week of hospitalisation. Our study revealed several significant findings in paediatric haematology/oncology patients, including *P. aeruginosa* as a major bacteriuria pathogen, the presence of ESBL isolates, and the emergence of carbapenem-resistant *P. aeruginosa* later during hospitalisation, requiring specific antimicrobial treatment if necessary. Additionally, symptomatic bacteriuria was more common in neutropenic patients. However, because of the small number of symptomatic patients, further studies are needed to clarify the importance of including urine culture in the diagnostic process for individuals with febrile neutropenia. It is crucial to note that the antimicrobial susceptibility data at the hospital and national levels may not align with the circumstances of a specific paediatric oncology or haematology ward. Consequently, monitoring of pathogen distribution and susceptibility on these wards is necessary when selecting an empirical antimicrobial treatment for UTI in paediatric haematology/oncology patients, and the empirical treatment should be modified accordingly.

## Figures and Tables

**Table 1:** Characteristics of study patients with bacteriuria (n = 74).

Characteristics	n (%)
Male, n (%)	35 (47.3)
Female, n (%)	39 (52.7)
Median age, years (range) (y)	8 (0–17)
Neutropenia (×10^9^/L)	20 (27.0)
Hematologic disease, n (%)	
Acute lymphoblastic leukemia	45 (60.8)
Acute myeloid leukemia	3 (4.1)
Non-Hodgkin and nonspecified lymphoma	5 (6.8)
Hodgkin lymphoma	6 (8.1)
Leukaemia, nonspecified	3 (4.1)
Myelodysplastic syndrome	1 (1.4)
Solid tumour, n (%)	
Neuroblastoma	4 (5.4)
Retinoblastoma	1 (1.4)
Hepatoblastoma	1 (1.4)
Sarcoma	2 (2.7)
Teratoma	1 (1.4)
Nasopharyngeal carcinoma	1 (1.4)
Malignant tumor of the sphenoidal sinus	1 (1.4)
Number of bacteriuria episodes per patient, n (%)	
1 episode	37 (50.0)
2 episodes	20 (27.0)
3 episodes	7 (9.5)
4 episodes	6 (8.1)
5 episodes	3 (4.1)
6 episodes	1 (1.4)

**Table 2:** Comparison of symptomatic and asymptomatic bacteriuria episodes (n = 143).

	Overall (n = 143)	Symptomatic Bacteriuria (n = 37)	Asymptomatic Bacteriuria (n = 106)	*p*
Female, n (%)	74 (51.8)	19 (51.4)	55 (51.9)	0.9554
Underlying disease, n (%)				
Solid tumour	21 (14.7)	7 (18.9)	14 (13.2)	0.3997
Hematologic disease	122 (85.3)	30 (81.1)	92 (86.8)	
History of UTI ^1^, n (%)	74 (51.8)	21 (56.8)	53 (50.0)	0.4804
Urinary catheter, n (%)	10 (7.0)	4 (10.8)	6 (5.7)	0.2919
Neutropenia, n (%)	25 (17.5)	11 (29.7)	14 (13.2)	0.0232
Causative pathogens, n (%)				
*Escherichia coli*	44 (30.8)	8 (21.6)	36 (34.0)	
*Klebsiella* spp.	27 (18.9)	11 (29.7)	16 (15.1)	
*Pseudomonas aeruginosa*	18 (12.6)	3 (8.1)	15 (14.2)	
*Proteus mirabilis*	11 (7.7)	4 (10.8)	7 (6.6)	
*Enterobacter cloacae*	9 (6.3)	1 (2.7)	8 (7.6)	0.1641
*Citrobacter* spp.	5 (3.5)	1 (2.7)	4 (3.8)	
*Acinetobacter junii*	1 (0.7)	0	1 (0.9)	
*Enterococcus* spp.	19 (13.3)	4 (10.8)	15 (14.2)	
polymicrobial bacteriuria	9 (6.3)	5 (13.5)	4 (3.8)	

^1^ UTI, urinary tract infection.

**Table 3:** Pathogens isolated from patients with bacteriuria episodes (n = 152).

Pathogen	n	%
*Escherichia coli*	49	32.2
*Klebsiella* spp.	34	22.4
*Pseudomonas aeruginosa*	22	14.5
*Enterococcus* spp.	21	13.8
*Proteus mirabilis*	11	7.2
*Enterobacter cloacae*	9	5.9
*Citrobacter* spp.	5	3.3
*Acinetobacter junii*	1	0.7

**Table 4:** Time of isolation of specific bacterial pathogens during hospitalization.

Pathogen	Mean (Days)	Range
*Escherichia coli* (n = 49)	8	0–94
*Klebsiella* spp. (n = 34)	28.5	0–162
*Pseudomonas aeruginosa* (n = 22)	17.5	0–78
*Enterococcus* spp. (n = 21)	7	0–51
*Proteus mirabilis* (n = 11)	10	0–65
*Enterobacter cloacae* (n = 9)	21	0–41
*Citrobacter* spp. (n = 5)	1	0–3
*Acinetobacter junii* (n = 1)	69	/

**Table 5:** Antimicrobial resistance rates in Gram-negative bacterial isolates (%).

	AMP	AMC	CXM	FEP	TZP	GM	AN	CIP	IMP	MEM	SXT
*Escherichia coli* (n = 49)	79.6	0	8.2	6.1	0	10.2	2	26.5	0	0	73.5
*Klebsiella* spp. (n = 34)	100	11.8	23.5	14.7	14.7	20.6	0	23.5	0	0	47.1
*Pseudomonas aeruginosa* (n = 22)	N/A	N/A	N/A	0	4.6	N/A	0	9.1	9.1	9.1	N/A
*Proteus mirabilis* (n = 11)	72.7	0	0	0	0	36.4	0	27.3	0	0	54.6
*Enterobacter cloacae* (n = 9)	100	100	100	22.2	33.3	22.2	0	11.1	0	0	33.3
*Citrobacter* spp. (n = 5)	100	100	100	20.0	0	0	0	20.0	0	0	60.0
*Acinetobacter junii* (n = 1)	N/A	N/A	N/A	N/A	0	100	0	0	0	0	N/A
All Gram-negative bacteria (n = 131)	88.0 (95/108)	16.7 (18/108)	24.1 (26/108)	8.5 (11/130)	6.9 (9/131)	17.4 (19/109)	0.8 (1/131)	21.4 (28/131)	1.5 (2/131)	1.5 (2/131)	59.3 (64/108)

AMP, ampicillin; AMC, co-amoxiclav; CXM, cefuroxime; FEP, cefepime; TZP, piperacillin/tazobactam; GM, gentamicin; AN, amikacin; CIP, ciprofloxacin; IMP, imipenem; MEM, meropenem; SXT, sulfamethoxazole/trimethoprim; N/A, bacteria-antimicrobial agent combination is not included in European Committee for Antimicrobial Susceptibility Testing (EUCAST) or Clinical and Laboratory Standards Institute (CLSI) standards.

## Data Availability

Data are contained within the article.
